# Assaying the effect of yeasts on growth of fungi associated with disease

**DOI:** 10.1186/s12866-020-01942-0

**Published:** 2020-10-21

**Authors:** Enikő Horváth, Matthias Sipiczki, Hajnalka Csoma, Ida Miklós

**Affiliations:** Department of Genetics and Applied Microbiology, Faculty of Science and Technology, University of Debrecen, Debrecen, Egyetem tér 1 4032 Hungary

**Keywords:** Disease-associated fungi, Bioactive agents, Yeast, Antagonism, *Metschnikowia* sp., Non-*albicans Candida* species, Natural resistance

## Abstract

**Background:**

Pathogenic fungi often cause serious infections mainly in immunocompromised persons. The number of infections caused by the non-*albicans Candida* or other species has significantly increased over the last years. These infections present a major challenge in the health sector because these pathogenic fungi have strong virulence and often show resistance to the commonly used antifungal treatments. To solve the problems caused by the drug resistant pathogenic fungi, it is necessary to find new antifungal agents and their sources. The aim of this study was to give evidence that yeasts can effectively fight against strains which belong to pathogenic fungi and reveal those yeasts which are able to inhibit growth of *Kodamaea ohmeri, Pichia kudriavzevii, Naganishia albida* or *Candida tropicalis.* Furthermore, we wanted to determine the effects of certain culturing factors on the growth inhibition.

**Results:**

Our screening revealed that although the strains belonging to pathogenic species were much more tolerant to the yeast-produced bioactive agents than the non-disease-associated yeasts, growth of *Kodamaea ohmeri* and *Candida tropicalis* could be inhibited by *Metschnikowia andauensis*, while *Naganishia albida* could be controlled by *Pichia anomala* or *Candida tropicalis.* Our data proved that the experimental circumstances could have a serious impact on the inhibitory capacity of the yeasts. Appearance of inhibition strongly depended on media, pH and temperature. Our data also shed some light on the fact that *Pichia kudriavzevii* must have high natural resistance to the yeast-produced agents, while other species, such as *Saccharomycopsis crataegensis* belonged to the easily inhibitable species.

**Conclusions:**

Our study suggests that yeast-produced bioactive agents could be potential growth inhibitory agents against the disease-associated fungi and yeasts can also contribute to alternative approaches to combat against pathogenic fungi. Our data revealed an important role of the culturing factors in inhibition and pointed to the complex nature of antagonism.

## Background

Fungaemia is associated with substantial morbidity and mortality of immunocompromised persons. Studies have demonstrated that fungal sepsis can quite often be caused by non-*albicans Candida* or other species, such as *Candida tropicalis, Pichia kudriavzevii* (synonyme *Candida krusei*), *Kodamaea ohmeri* (synonyme *Candida guillermondii*) cells or *Naganishia albida* (formerly *Cryptococcus albidus*) ([1, 2], reviewed in [3–9]). However, the most frequent species which can cause candidemia can depend on regions, age of the patients, or type of the medical interventions (reviewed in [10]). According to the reports, *Pichia kudriavzevi* cells were isolated from hematology-oncology services or neonatal care units in different geographic locations and it was supposed to be the fifth most common cause of candidemia [1, 2]. *Kodamaea ohmeri* cells were isolated from infant and neonate, from wound lesions and blood [reviewed in 3, 4, 5, 6]. *Candida tropicalis* is one of the most common colonizer in tropical countries [reviewed in 11]. Its infections can be found both in animals and humans and can cause gastrointestinal invasions or arthritis ([11], reviewed in [7, 12]). *Naganishia albida* was isolated from transplant recipients or lesions ([8], reviewed in [9]).

The major therapeutic challenges of the health sector arise from the resistance of these pathogenic fungi to the commonly used antifungal agents that greatly contribute to their survival and successful infections ([5, 11], reviewed in [7, 13]). Consequently, finding new inhibitory agents against these disease-associated fungi is becoming increasingly urgent.

Different studies have tried to find bioactive agents which can inhibit cell division or hyphal formation of these pathogenic fungi [14–16]. Mostly they wanted to find effective agents by screening new synthetic drugs or testing natural agents, such as antimycotic plant oils [14–16]. To solve the fungal resistance problem, a further possibility can be investigation and application of the yeasts having biocontrol capacity or the yeast-produced antifungal agents. Namely, yeast species are often able to reduce or inhibit growth of destructive microbes ([17, 18] reviewed in [19, 20]). Different mechanisms, such as competition for nutrients, or secretion of antifungal compounds have been proposed as being responsible for their antagonistic activity ([21], reviewed in [19, 20, 22]). They can produce siderophores, cell wall degrading enzymes or further unknown bioactive agents [23–29].

Based on the above, we hypothesized that yeasts can effectively fight also against disease-associated fungi. That is, in this study we wanted to find out whether cell division of the strains belonging to non-*albicans Candida* disease-associated fungi, such as *Candida tropicalis, Pichia kudriavzevii, Kodamaea ohmeri* or *Naganishia albida* could be inhibited by certain yeast species or not.

A further aim of ours was to find these inhibitory yeast species and influencing factors of the inhibition. To this end, type strains, strains isolated from nature, strains belonging to well-known antagonistic species and species not studied for biological control were equally tested.

Our screening provided further evidence for the antagonistic ability of yeasts, revealed those species which were able to inhibit cell division of the strains which belong to infectious fungi. The data proved that the appearance of inhibition can strongly depended on the media, pH and temperature. Our data also suggested that *Pichia kudriavzevii* must have strong inherited resistance to the yeast-produced antifungal agents.

## Results

### Growth of *Kodamaea ohmeri*, *Candida tropicalis* and *Naganishia albida* cells could be inhibited by yeast species

**Fig. 1:**
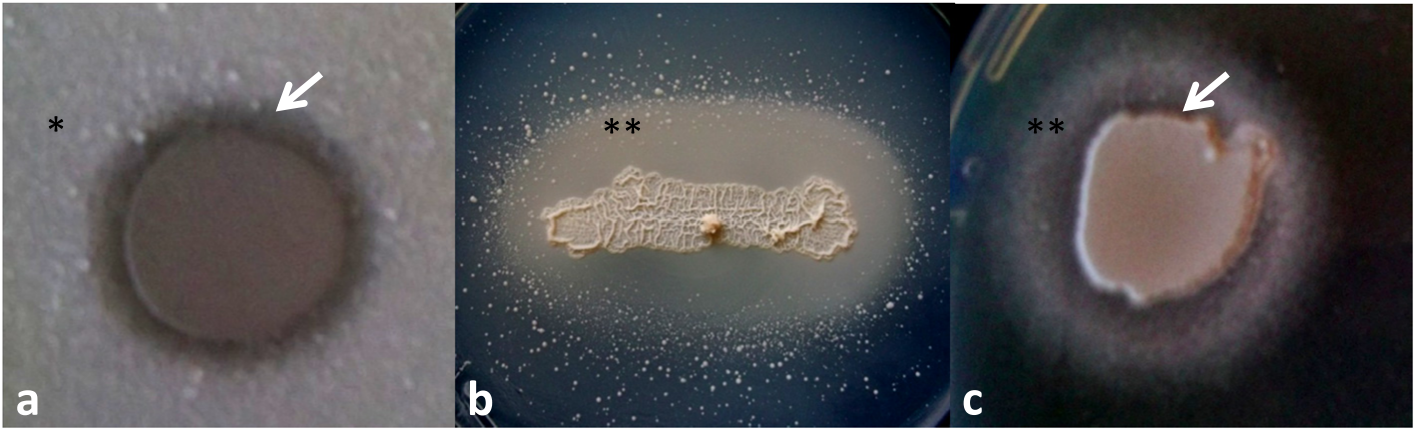
Yeast-produced bioactive agents can cause not only inhibition, but growth stimulation. **a** Inhibition - lawn: *Kodamae ohmeri* (11–466), test-strain: *Metschnikowia andauensis* (11–1120) (*C. tropicalis* lawn gave similar result). (White arrows show the clear inhibitory zone, where cells of the lawn could not divide. * indicates the growing cells of the lawn farther from the test-strain). **b** Growth stimulation - lawn: *Candida diversa* (11–470), test-strain: *Candida tropicalis* (11–476). ** indicates the growth stimulation. **c** Co-occurence of inhibitory- and stimulation zones-lawn: *Saccharomycopsis crataegensis* (11–463), test-strain: *Metschnikowia andauensis* (11–1120). EMMA media (pH 6.5) were incubated at room temperature and photographed after 5 days

In order to find yeast species which are able to inhibit cell division of the strains belonging to disease-associated species, several yeasts (test-strains) were investigated. Species with known and un-known biocontrol capacity were equally tested (Table 1). Our results showed that growth of *Kodamaea ohmeri* (Fig. 1a) and *Candida tropicalis* could be inhibited by *M. andauensis* cells, while *Naganishia albida* was controlled by *P. anomala* and *C. tropicalis* (Table 1)(indicated with +)*.* Other test-species, among them the well-known antagonistic species, such as *Metschnikowia pulcherrima* were not able to form an inhibitory zone on the lawn of the strains of disease-associated species (indicated with -), in turn they were effective in the case of several strains belonging to non-disease-associated species (Table 1). The *Saccharomycopsis crataegensis* and *Wickerhamomyces orientalis* species were especially sensitive. They were inhibitable by almost all the test-strains, while the *Pichia kudriavzevii* seemed to be rather resistant (Table 1). Interestingly, in some cases, growth stimulation of the lawn (indicated with S in the Table 1, Fig. 1b) or co-occurrence of inhibitory- and stimulation zones could also be detected (indicated with I-S in the Table 1, Fig. 1c).

**Table 1:** Yeasts are able to inhibit growth of strains belonging to disease-associated species

Test-strains		Lawn: diseases-associated species				Lawn: non-disease-associated species						
Collection number	Species with known biocontrol capacity	11–462 *Pichia kudriavzevii*	11–466 *Kodamaea ohmeri*	11–471 *Candida tropicalis*	2–1365 * Naganishia albida*	11–465 *Candida stigmatis*	11–463 *Saccharomycopsis crataegensis*	11–467 *Starmerella meliponinorum*	11–468 *Torulaspora delbrueckii*	11–469 *Candida citri*	11–470 *Candida diversa*	11–461 *Wickerhamomyces orientalis*
11–460	*Pichia kudriavzevii*	–	–	–	–	–	+	–	S	+	–	–
11–502	*Pichia anomala*	–	–	–	+	+	+	–	+	+	–	+
11–481	*Saccharomyces cerevisiae*	–	–	–	S	–	+	nd	+	–	–	+
11–476	*Candida tropicalis*	–	–	–	+	–	+	–	–	–	S^a^	–
11–505	*Pichia guilliermondii*	–	–	–	–	–	+	+	–	–	–	S
11–1120	*Metschnikowia andauensis*	–	+^a^	+	–	+	I-S^a^	+	+	+	+	+
11–578	*Metschnikowia pulcherrima*	–	–	–	S	+	S	+	–	+	+	+
11–11	*Metschnikowia pulcherrima*	–	–	–	S	+	S	–	–	–	+	+
	**Species with no known biocontrol capacity**											
11–465	*Candida stigmatis*	–	–	–	–	–	+	–	–	–	–	–
11–472	*Hanseniaspora thailandica*	–	–	–	S	–	+	–	–	–	–	+
11–473	*Candida ethanolica*	–	–	–	–	–	+	–	–	–	S	+
11–486	*Pichia dorogensis*	–	–	–	–	–	+	–	–	–	–	+
11–489	*Cryptococcus flavescens*	–	–	–	–	–	+	–	–	–	–	S
11–1055	*Candida verbasci*	–	–	–	–	–	+	+	–	–	–	+
11–461	*Wickerhamomyces orientalis*	**–**	**–**	**–**	**–**	**S**	**–**	**+**	**–**	**–**	**–**	**–**

+: presence of inhibitory zone on EMMA, pH 7, room temperature

-: absence of inhibitory zone

S: growth stimulation

I-S: co-occurence of inhibitory- and stimulation zones

nd: not determined

^a^demonstrated by photo (Fig.1)

### *Pichia kudriavzevii* has strong resistance

Our previous screening suggested that *Pichia kudriavzevii* could have strong resistance to the yeast-produced agents (Table 1). To learn whether this is true or not, further test-strains belonging to different species and originating from different regions of the world were investigated on the *Pichia kudriavzevii* lawn. Our data confirmed the strong resistance of *Pichia kudriavzevii* (Table 2), since a total of 50 strains belonging to 35 species were not able to inhibit its growth both on complete and minimal media (Table 2). In contrast, *Saccharomycopsis crataegensis* cells (used as control) could be inhibited by several yeast species (Table 2).

**Table 2:** *Pichia kudriavzevii* has strong resistance against yeast-produced bioactive agents

Test-strains				Inhibitory zone	
**Collection number**	**Species**	**Origin**	**Isolation source**	**11–460**^a^ ***Pichia kudriavzevii***	**11–463**^a^ ***Saccharomycopsis crataegensis***
				**Media**	
				**EMMA/YPGA**	**EMMA/YPGA**
11–502	*Pichia anomala*	Laos, Luang Prabang	flower	−/−	−/+
11–520	*Pichia anomala*	Laos, Vientiane	plant-louse	−/−	−/+
11–522	*Pichia anomala*	Laos, Vientiane	flower	−/−	−/+
11–485	*Pichia bruneiensis*	Borneo, Brunei	flower	−/−	−/+
11–480	*Pichia manshurica*	Philippines, Manila	banana	−/−	+/−
11–461	*Wickerhamomyces orientalis*	Sri Lanka, Galle	fruit	−/−	−/−
11–496	*Saccharomycopsis crataegensis*	Philippines, Manila	rotting fruit	−/−	−/−
11–464	*Metschnikowia koreensis*	India, Hyderabad	flower	−/−	−/−
11–482	*Metschnikowia koreensis*	Borneo, Brunei	flower	−/−	−/+
11–524	*Metschnikowia laotica*	Laos, Luang Prabang	fruit	−/−	−/+
11–1062	*Metschnikowia pulcherrima*	Georgia, Tbilisi	fruit	−/−	−/+
11–523	*Candida glabrata*	Laos, Vientiane	flower	−/−	+/−
11–484	*Candida boidinii*	Borneo, Brunei	flower	−/−	−/−
11–471	*Candida tropicalis*	Philippines, Caticlan	banana	−/−	−/−
11–521	*Candida tropicalis*	Laos, Vientiane	mushroom	−/−	−/+
11–470	*Candida diversa*	Borneo, Brunei	mango	−/−	+/−
11–477	*Candida californica*	Borneo, Brunei	fruit	−/−	−/−
11–478	*Candida californica*	Borneo, Brunei	fruit	−/−	−/−
11–473	*Candida ethanolica*	Borneo, Brunei	papaya	−/−	−/−
11–469	*Candida citri*	Borneo, Brunei	lemon	−/−	−/+
11–488	*Candida pseudointermedia*	Borneo, Brunei	flower	−/−	−/+
11–479	*Candida zemplinina*	Philippines, Manila	banana	−/−	−/−
11–487	*Candida borneonana*	Borneo, Brunei	rotting fruit	−/−	−/−
11–504	*Candida intermedia*	Laos, Luang Prabang	flower	−/−	−/+
11–506	*Candida jaroonii*	Laos, Luang Prabang	rotting fruit	−/−	−/+
11–512	*Candida jaroonii*	Laos, Luang Prabang	flower	−/−	−/+
11–514	*Candida jaroonii*	Laos, Luang Prabang	flower	−/−	−/+
11–507	*Candida suratensis*	Laos, Luang Prabang	rotting fruit	−/−	−/+
11–509	*Candida suratensis*	Laos, Luang Prabang	fruit	−/−	−/+
11–510	*Candida suratensis*	Laos, Luang Prabang	fruit	−/−	−/+
11–513	*Candida butyri*	Laos, Luang Prabang	flower	−/−	–
11–517	*Candida sergipensis*	Laos, Vientiane	leaf	−/−	+/−
11–519	*Candida parapsilosis*	Laos, Vientiane	plant-louse	−/−	−/−
11–466	*Kodamaea ohmeri*	India, Hyderabad	flower	−/−	−/+
11–490	*Kodamaea ohmeri*	Philippines, Manila	fruit	−/−	−/+
11–500	*Kodamaea ohmeri*	Philippines, Manila	fruit	−/−	−/+
11–467	*Starmerella meliponinorum*	India Hyderabad	flower	−/−	+/−
11–1071	*Starmerella caucasica*	Azerbaijan, Baku	flower	−/−	+/−
11–474	*Torulaspora delbrueckii*	Borneo, Brunei	papaya	−/−	+/−
11–475	*Issatchenkia terricola*	Borneo, Brunei	lemon	−/−	−/−
11–491	*Hanseniaspora thailandica*	Philippines, Manila	rotting fruit	−/−	−/−
11–495	*Hanseniaspora thailandica*	Philippines, Manila	rotting fruit	−/−	−/−
11–499	*Hanseniaspora thailandica*	Philippines, Manila	rotting fruit	−/−	−/−
11–494	*Hanseniaspora uvarum*	Philippines, Manila	papaya	−/−	−/−
11–501	*Aureobasidium pullulans*	Philippines, Manila	fruit	−/−	−/+
11–511	*Metahyphopichia laotica*	Laos, Luang Prabang	fruit	−/−	−/−
11–516	*Metahyphopichia laotica*	Laos, Vientiane	flower	−/−	−/+
11–518	*Cryptococcus heveanensis*	Laos, Vientiane	flower	−/−	−/−
11–489	*Cryptococcus flavescens*	Philippines, Banaue	fruit	−/−	+/−

Petri dishes were incubated at room temperature

+: presence of inhibitory zone

-: absence inhibitory zone

^a^11–460 and 11–463 species used as lawn were isolated from Sri Lanka, Colombo

### Influencing factors of the growth inhibition

**Fig. 2:**
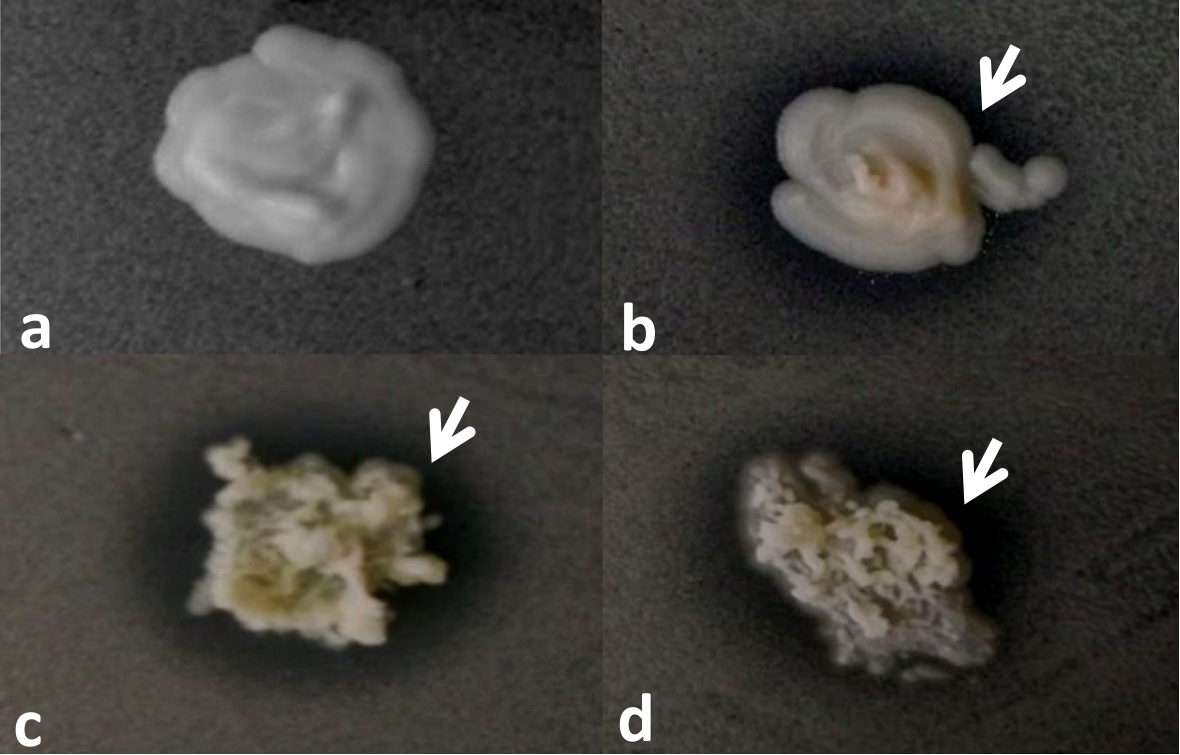
Effect of pH value on growth inhibition. Growth of *Naganishia albida* (2–1365) was investigated on EMMA medium (pH 5 and 6.5) in the presence of test-strains *Pichia anomala* (11–502) and *Trichosporon asahii* (11–1127). The Petri dishes were incubated at 24 °C for 3–10 days. **a**: *Pichia anomala*, pH 5, **b**: *Pichia anomala,* pH 6.5, **c**: *Trichosporon asahii*, pH 5 (d): *Trichosporon asahii,* pH 6.5. **a**: absence of inhibitory zone, (**b, c, d**): presence of inhibitory zone. White arrows show the clear inhibitory zone, where cells of the *Naganishia albida* (lawn) could not divide

Our earlier observation (Table 2-*Saccharomycopsis crataegensis*) and previous studies [30, 31] have suggested that medium and culture conditions can have a strong impact on biocontrol activity. Thus, we repeated our experiments with one of the disease-associated species (*Naganishia albida*) applying minimal (EMMA) and complete (YPA) media, different pH, temperature and using further test-strains. Our data confirmed that culture conditions can strongly influence antagonistic effect of the test-strains and optimal conditions of antagonism can be species-specific (Table 3). Changes of the pH value or medium influenced the growth inhibition differently in the case of the different species (Fig. 2, Table 3). There was a less sensitive strain (*Candida insectorum*), a strain which showed inhibitory capacity only at lower pH, such as *Pichia dorogensis,* while the appearance of antagonism depended on the media in the case of *Trichosporon asahii* or *Sporidiobolus ruineniae* (Table 3)*.* Inhibitory capacity of *Wickerhamomyces orientalis* seemed to be influenced by temperature (Table 3). Modification of the culture factors could lead to finding further antagonistic species against *Naganishia albida* (e.g. *Candida insectorum* or *Pichia dorogensis*)(Table 3).

**Table 3:** Influencing factors of the growth inhibition. Alteration of media, pH and temperature allowed us to identify further antagonistic species against *Naganishia albida*

Test-strains		Lawn: 2–1365 ***Naganishia albida***							
Collection number	Species	pH = 5 YPGA 24 °C	pH = 6.5 YPGA 24 °C	pH = 5 EMMA 24 °C	pH = 6.5 EMMA 24 °C	pH = 5 YPGA 30 °C	pH = 6.5 YPGA 30 °C	pH = 5 EMMA 30 °C	pH = 6.5 EMMA 30 °C
11–460	*Pichia kudriavzevii*	–	–	–	–	–	–	+	–
11–1146	*Pichia kudriavzevii*	–	–	–	–	–	–	+	–
11–502	*Pichia anomala*	+	+	-^a^	+^a^	+	+	–	+
11–481	*Saccharomyces cerevisiae*	–	–	–	S	–	–	+	–
11–476	*Candida tropicalis*	+	+	–	+	+	+	–	+
11–505	*Pichia guilliermondi*	–	–	–	–	–	–	–	–
11–1120	*Metschnikowia andauensis*	–	–	I-S	–	+	–	+	–
11–578	*Metschnikowia pulcherrima*	–	–	S	–	–	–	S	–
11–11	*Metschnikowia pulcherrima*	–	–	S	S		–	S	–
11–465	*Candida stigmatis*	–	–	–	–	–	–	–	–
11–472	*Hanseniaspora thailandica*	–	–	–	S	–	–	–	–
11–473	*Candida ethanolica*	–	–	–	–	–	–	–	–
11–486	*Pichia dorogensis*	+	–	+	–	+	–	+	–
11–489	*Cryptococcus flavescens*	–	–	–	–	–	–	–	–
11–1055	*Candida verbasci*	–	–	–	–	–	–	–	–
11–461	*Wickerhamomyces orientalis*	+	+	–	–	–	–	–	–
11–523	*Candida glabrata*	–	–	–	–	–	–	–	–
11–1127	*Trichosporon asahii*	–	–	+^a^	+^a^	–	–	+	+
11–1135	*Pichia kluyveri*	–	–	–	–	–	–	–	–
11–1185	*Sporidiobolus ruineniae*	+	+	–	–	+	+	–	S
11–1193	*Candida insectorum*	+	+	+	+	+	+	+	+
2–1366	*Candida magnifica*	–	–	–	–	–	–	–	–

+: presence of the inhibitory zone

-: absence of the inhibitory zone

nd: not determined,

S: stimulation

I-S: co-occurence of inhibitory- and stimulation zone

^a^: demonstrated by photo (Fig. 2)

## Discussion

Non-*albicans Candida* or other species, such as *Naganishia albida* are frequently isolated from hospitalized persons [1–13]. A major issue in the health sector is that these species often show resistance to the commonly used antifungal treatments [2, 5, 7, 13]. The consequences of these fungal infections can be very serious, especially in children, neonates or immunocompromised patients. Thus, finding new inhibitory agents and their possible sources is becoming increasingly urgent.

Earlier studies have observed that antagonistic interaction can occur between yeasts species and they can regulate each other’s growth [17, 19–24, 32]. Antagonistic yeasts have mainly been investigated against postharvest pathogens of fruits and vegetables [17, 19–24, 32] and only a small number of data suggest that yeasts could also inhibit fungi associated with disease [18]. Based on the above, the main focus of this study was to reveal those yeasts which were able to inhibit growth of strains which belong to pathogenic species. Type strains, strains isolated from nature, species with known and un-known antagonistic capacity were equally tested against the medically important fungi. Our data revealed that growth of *Kodamaea ohmeri* and *Candida tropicalis* could be inhibited by *Metschnikowia andauensis*, while *Naganishia albida* could be controlled by *Pichia anomala* and *Candida tropicalis*. These results are in good agreement with the experimental data of Kunyeit, who has demonstrated an inhibitory effect of the probiotic yeasts [18]. Our data also confirm that yeasts can be promising means of the fight against pathogenic fungi, can influence future trends of antimicrobial treatment and they can be sources of new antifungal agents.

However, we have to notice that the strains belonging to pathogenic fungi were somehow much more tolerant to the yeast-produced bioactive agents than the non-disease-associated yeasts, such as e.g. *Saccharomycopsis crataegensis* and *Wickerhamomyces orientalis.* One of them, *Pichia kudriavzevii* was especially tolerant to yeast-produced bioactive agents, because we failed to find any inhibitory yeast against it after application of 50 different test-strains (belonging to 35 species). Causes of its high resistance are not known and require further study. We suppose that it can be an inherited natural species-specific feature of *Pichia kudriavzevii*, because our strain was isolated from nature and had not previously come into contact with antifungal medicaments. Its high natural tolerance might be related to the multidrug resistance found in the clinical isolates [2].

Antagonism can be attributed to different features, such as competition for nutrients, or secretion of antifungal compounds ([21], reviewed in [19, 20, 22]). However, enzymes and proteins produced by antagonistic yeasts are partly known [33–37], we do not know exactly which inhibitory agents of our yeasts investigated were effective against the strains belonging to the disease-associated species. Further studies are required to identify these yeast-produced drugs. We assume that majority of the inhibitory test-strains might produce different bioactive agents because their antagonistic capacity was mostly influenced by different experimental factors. In certain cases the complete medium, while in other cases the lower pH favored the appearance of inhibition. These results drew our attention that optimal conditions of antagonism can be species-specific and its appearance strongly depends on the partner microbes. These results might be supported by data obtained with antagonistic yeasts used in postharvest disease control of fruits [30, 31]. Studying of the culture factors was useful because it could lead to finding further inhibitory strains against *Naganishia albida* and suggested that antagonistic tests are worth to carry out under different circumstances. Our data also shed some light on the complexity of the interaction between yeasts, because, besides inhibition, growth stimulation or co-appearance of inhibitory- and stimulation zones were also noticed, similarly to other species [38].

Taken together, this study provides further evidence that certain yeast species can be good candidates for finding those new bioactive agents that can be suitable to inhibit cell division of the disease-associated fungi. The results also drew our attention to the important role of culture conditions in antagonism.

## Conclusions

The significance of this study is that it has revealed those yeast species which are able to inhibit growth of *Naganishia albida, Kodamaea ohmeri, Candida tropicalis* strains, whose number is increasing in the isolates originating from hospitalized persons. Our data give evidence that certain yeast species might be good basics of new alternative approaches to combat fungal infections. Since our data pointed to the important role of certain culture factors on inhibition, the complex nature of yeast-yeast interaction and high natural resistance of the *Pichia kudriavzevii*, they can contribute to the precise development of experimental conditions of future studies.

## Methods

### Origin of the strains and yeast isolation

Strains used in this study were collected by Prof. Sipiczki from different regions of the world, except for three type-strains, *Metschnikowia andauensis* (11–1120, HA 1657) and *Metschnikowia pulcherrima* (11–11, CBS 610, ATCC 22032) (11–578, CBS 5833, ATCC 18406) which were purchased from collections. The collected samples originated from fruits or flowers (Table 2), because we wanted to investigate such strains which had not previously come into contact with antifungal medicaments. The fruits and flowers were dissected and samples were taken under aseptic conditions. The samples were put in sterile water and aliquots were spread onto YPA medium. The agar plates were incubated at 25 °C for 7 days. Single yeast colonies were isolated under sterile circumstances. Phase-contrast microscopy (Olympus BX40) was used to check cell morphology. The isolated strains were stored at -80 °C until taxonomic and further tests.

### Determination of taxonomic position of the strains

Taxonomic positions of the collected yeast strains were identified by PCR and sequencing methods. D1/D2 domains of 26S rDNA genes were amplified with primers NL1 (5′-GCA TAT CAA TAA GCG GAG GAA AAG-3′) and NL4 (5′-GGTCCG TGT TTC AAG ACG G-3′) [39]. PCR parameters were: 94 °C 2 min, 95 °C 1 min, 51 °C 1 min, 72 °C 1 min, (30X) 72 °C 10 min. The PCR products were purified and sequenced using the same primers. NCBI database (https://blast.ncbi.nlm.nih.gov/Blast) was used for the sequence analyses. The taxonomic positions of the strains were accepted when 100% identity was found to the corresponding sequences of the type-strains deposited in the databases (Fig. S1). Since the strains listed in Tables 1, 2, 3 belonged to known species their sequences were not deposited in a database.

### Culture media and standard yeast culture conditions

Generally, yeasts were cultured on Yeast Extract Agar (YEA) medium (1% yeast extract-VWR J850, 2% glucose-Fluca 49,159, 2% agar-Sigma 0540) and incubated at 25 °C.

For spot assays the inoculum was prepared from cells of a single colony. The cells of the pre- and main cultures were grown in Yeast Peptone Glucose medium (YPG) (1% yeast extract, 2% peptone-VWR 84610, 2% glucose) for overnight at 28 °C in a shaker. Spot assays were carried out on YPGA (YPG + 2.5% agar) and Edinburgh Minimal Medium (EMMA) [39].

### Spot assay to monitor growth inhibition

The cells of the disease-associated fungi cultured overnight in YPG, at 28 °C, in a shaker, were harvested, washed with sterile distilled water and cell suspension was prepared in sterile water (final cell density was 7 × 10^7^ cell/ml). EMMA minimal and YPGA complete media were flooded with 1 mL of the cell suspension (we called it lawn). When the surface of the plates dried in a sterile box, the yeast strains to be tested for antagonistic capacity (we called it test-strain) were streaked or dropped (10ul of 7X10^7^ cell/ml cell suspension) onto the centre of the agar plates (Figs. 1, 2, S2). The Petri dishes were incubated at the indicated temperatures.

Appearance of the clear inhibitory zones (Fig. S2a) was investigated after 3–10 days. When cells of the lawn were not able to grow around the test-strain, while they showed at the same time normal growth without or far from the test-strain -see Fig. 2b,c,d, S2a (and the zone was similar to the inhibitory zones produced by *Metschnikowia pulcherrima* type strains on the *Candida stigmatis* lawn), it was indicated with (+) because of the presence of inhibitory zone (see in the Tables). When cells of the lawn were able to grow around the test-strain and showed similar growth as in absence of the test-strain or far from it (see Fig. 2a, Fig. S2b), it was indicated with (−) because of the absence of inhibitory zone (see in the Tables). The lawns were always prepared at the same time, on the same media and were also compared to each other. The results come from three or more separate experiments.

### The influencing factors of growth inhibition

To learn the effect of pH, temperature and composition of the media on the growth inhibition, the spot assays were repeated using EMMA and YPGA media. Their pH values were set to 5 and 6.5. We used these two pH values, because earlier data suggested that antagonistic capacity of several yeast species was similarly at pH 5.0–5.5 and 6–6.5-7 [35]. The Petri dishes were incubated at 24 and 30 °C because lower or higher temperatures did not favour the cell division of several species (data not shown). The temperatures of the incubators were checked with thermometer.

### Grouping of the strains for growth inhibition assay

One group of the strains was called "test-strain” and their antimicrobial capacity was investigated. This group contained species with known biocontrol capacity (*Pichia anomala*, *Metschnikowia andauensis*, *Metschnikowia pulcherrima*, *Saccharomyces cerevisiae*) and species which were randomly selected from those yeasts whose biocontrol capacity was not earlier investigated (*Candida stigmatis*, *Hanseniaspora thailandica*, *Candida ethanolica*, *Pichia dorogensis*, *Cryptococcus flavescens*, *Candida verbasci, Wickerhamomyces orientalis*) (Table 1).

The other group of the species was used as "lawn”. The strains belonging to disease–associated species (*Pichia kudriavzevii, Kodamaea ohmeri, Candida tropicalis, Naganishia albida*) were investigated for growth inhibition (Table 1). Besides the strains which belong to disease–associated species, non-disease related species, such as *Saccharomycopsis crataegensis, Starmerella meliponinorum* etc. were also tested to reveal whether there is any difference in their sensitivity compared to the yeasts associated with disease. The non-disease-associated yeast strains were used as lawn, because our preliminary data suggested that they might be more sensitive than the strains belonging to disease–associated species.

## Supplementary information

**Additional file 1 Figure S1.** BLAST analysis of the nucleotide sequence obtained from 11-473 strain. 100% identity was found to the *Candida ethanolica* ribosomal DNA (https://blast.ncbi.nlm.nih.gov/Blast). Similar results were obtained in the case of the other strains used in this study. Query: nucleotide sequence of 11–473 strain. Sbjct: nucleotide sequence of *Candida ethanolica* type-strain.

**Additional file 2 Figure S2.** General arrangement of a spot assay to monitor growth inhibition of the test-strains. (a) presence of the inhibitory zone (white arrow shows the clear inhibitory zone, where the cells were not able to grow around the test-strain in contrast to the distal parts of the lawn. (b) absence of the inhibitory zone. The lawns were prepared at the same time on the same media. (A) and (B) indicate the species which were used as test-strains. (C) indicates the species which was used as lawn.

## Data Availability

All data generated or analysed during this study are included in this published article and its supplementary information files.
