# Supplementary material for: Assaying the effect of yeasts on growth of fungi associated with disease
Source: BMC Microbiol. 2020 Oct 21;20:320. doi: 10.1186/s12866-020-01942-0 (PMC7579944; doi:10.1186/s12866-020-01942-0)
Supplement: Supplementary file 1 — Additional file 1 Figure S1. BLAST analysis of the nucleotide sequence obtained from 11-473 strain. 100% identity was found to the Candida ethanolica ribosomal DNA (https://blast.ncbi.nlm.nih.gov/Blast). Similar results were obtained in the case of the other strains used in this study. Query: nucleotide sequence of 11–473 strain. Sbjct: nucleotide sequence of Candida ethanolica type-strain. [file 12866_2020_1942_MOESM1_ESM.pptx]

## Slide 1
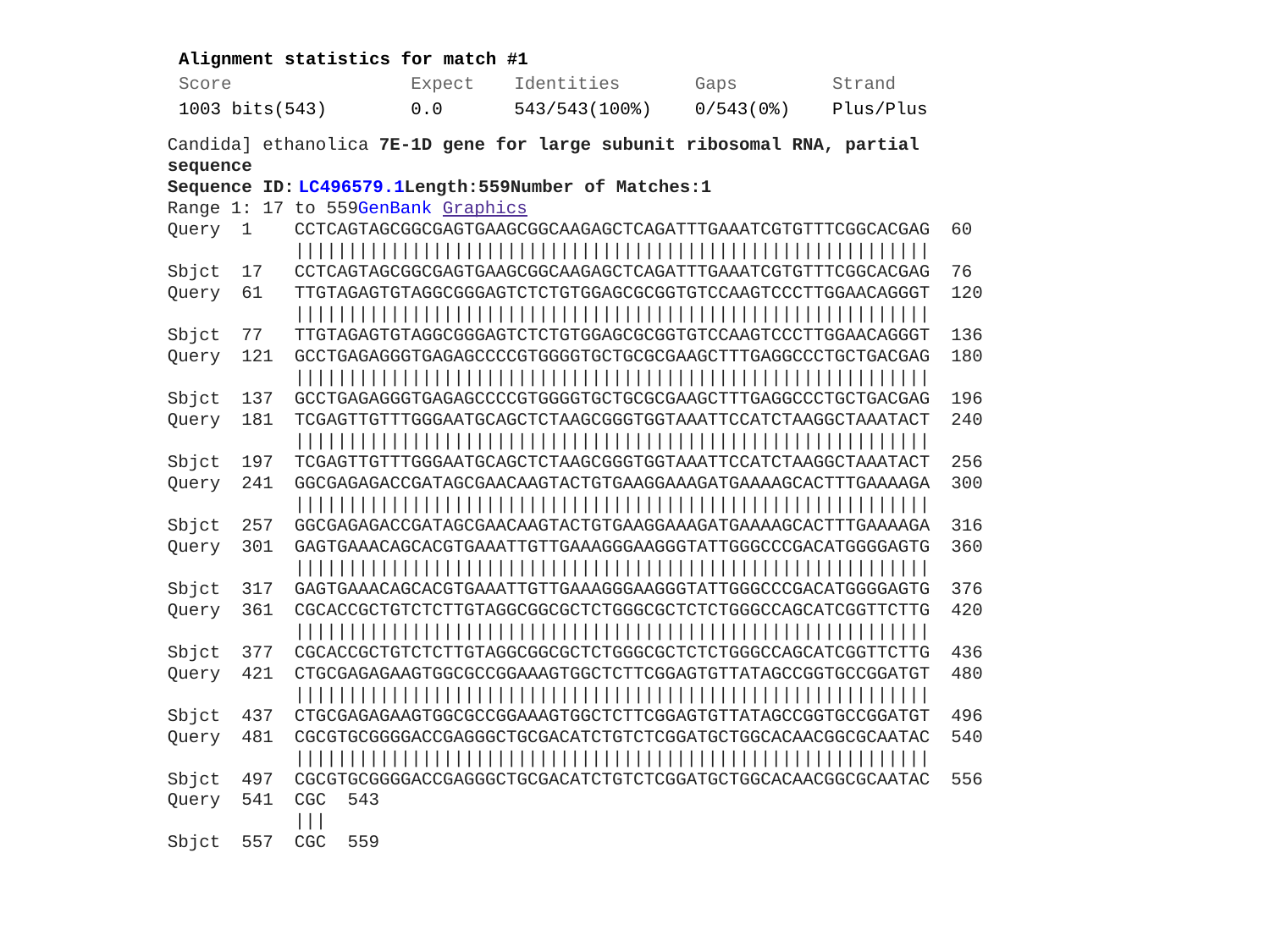

| Alignment statistics for match #1 | | | | |
| --- | --- | --- | --- | --- |
| Score | Expect | Identities | Gaps | Strand |
| 1003 bits(543) | 0.0 | 543/543(100%) | 0/543(0%) | Plus/Plus |
Candida] ethanolica 7E-1D gene for large subunit ribosomal RNA, partial sequence
Sequence ID: LC496579.1Length:559Number of Matches:1
Range 1: 17 to 559GenBank Graphics
Query 1 CCTCAGTAGCGGCGAGTGAAGCGGCAAGAGCTCAGATTTGAAATCGTGTTTCGGCACGAG 60
 ||||||||||||||||||||||||||||||||||||||||||||||||||||||||||||
Sbjct 17 CCTCAGTAGCGGCGAGTGAAGCGGCAAGAGCTCAGATTTGAAATCGTGTTTCGGCACGAG 76
Query 61 TTGTAGAGTGTAGGCGGGAGTCTCTGTGGAGCGCGGTGTCCAAGTCCCTTGGAACAGGGT 120
 ||||||||||||||||||||||||||||||||||||||||||||||||||||||||||||
Sbjct 77 TTGTAGAGTGTAGGCGGGAGTCTCTGTGGAGCGCGGTGTCCAAGTCCCTTGGAACAGGGT 136
Query 121 GCCTGAGAGGGTGAGAGCCCCGTGGGGTGCTGCGCGAAGCTTTGAGGCCCTGCTGACGAG 180
 ||||||||||||||||||||||||||||||||||||||||||||||||||||||||||||
Sbjct 137 GCCTGAGAGGGTGAGAGCCCCGTGGGGTGCTGCGCGAAGCTTTGAGGCCCTGCTGACGAG 196
Query 181 TCGAGTTGTTTGGGAATGCAGCTCTAAGCGGGTGGTAAATTCCATCTAAGGCTAAATACT 240
 ||||||||||||||||||||||||||||||||||||||||||||||||||||||||||||
Sbjct 197 TCGAGTTGTTTGGGAATGCAGCTCTAAGCGGGTGGTAAATTCCATCTAAGGCTAAATACT 256
Query 241 GGCGAGAGACCGATAGCGAACAAGTACTGTGAAGGAAAGATGAAAAGCACTTTGAAAAGA 300
 ||||||||||||||||||||||||||||||||||||||||||||||||||||||||||||
Sbjct 257 GGCGAGAGACCGATAGCGAACAAGTACTGTGAAGGAAAGATGAAAAGCACTTTGAAAAGA 316
Query 301 GAGTGAAACAGCACGTGAAATTGTTGAAAGGGAAGGGTATTGGGCCCGACATGGGGAGTG 360
 ||||||||||||||||||||||||||||||||||||||||||||||||||||||||||||
Sbjct 317 GAGTGAAACAGCACGTGAAATTGTTGAAAGGGAAGGGTATTGGGCCCGACATGGGGAGTG 376
Query 361 CGCACCGCTGTCTCTTGTAGGCGGCGCTCTGGGCGCTCTCTGGGCCAGCATCGGTTCTTG 420
 ||||||||||||||||||||||||||||||||||||||||||||||||||||||||||||
Sbjct 377 CGCACCGCTGTCTCTTGTAGGCGGCGCTCTGGGCGCTCTCTGGGCCAGCATCGGTTCTTG 436
Query 421 CTGCGAGAGAAGTGGCGCCGGAAAGTGGCTCTTCGGAGTGTTATAGCCGGTGCCGGATGT 480
 ||||||||||||||||||||||||||||||||||||||||||||||||||||||||||||
Sbjct 437 CTGCGAGAGAAGTGGCGCCGGAAAGTGGCTCTTCGGAGTGTTATAGCCGGTGCCGGATGT 496
Query 481 CGCGTGCGGGGACCGAGGGCTGCGACATCTGTCTCGGATGCTGGCACAACGGCGCAATAC 540
 ||||||||||||||||||||||||||||||||||||||||||||||||||||||||||||
Sbjct 497 CGCGTGCGGGGACCGAGGGCTGCGACATCTGTCTCGGATGCTGGCACAACGGCGCAATAC 556
Query 541 CGC 543
 |||
Sbjct 557 CGC 559
