# Supplementary material for: Assaying the effect of yeasts on growth of fungi associated with disease
Source: BMC Microbiol. 2020 Oct 21;20:320. doi: 10.1186/s12866-020-01942-0 (PMC7579944; doi:10.1186/s12866-020-01942-0)
Supplement: Supplementary file 2 — Additional file 2 Figure S2. General arrangement of a spot assay to monitor growth inhibition of the test-strains. (a) presence of the inhibitory zone (white arrow shows the clear inhibitory zone, where the cells were not able to grow around the test-strain in contrast to the distal parts of the lawn. (b) absence of the inhibitory zone. The lawns were prepared at the same time on the same media. (A) and (B) indicate the species which were used as test-strains. (C) indicates the species which was used as lawn. [file 12866_2020_1942_MOESM2_ESM.pptx]

## Slide 1
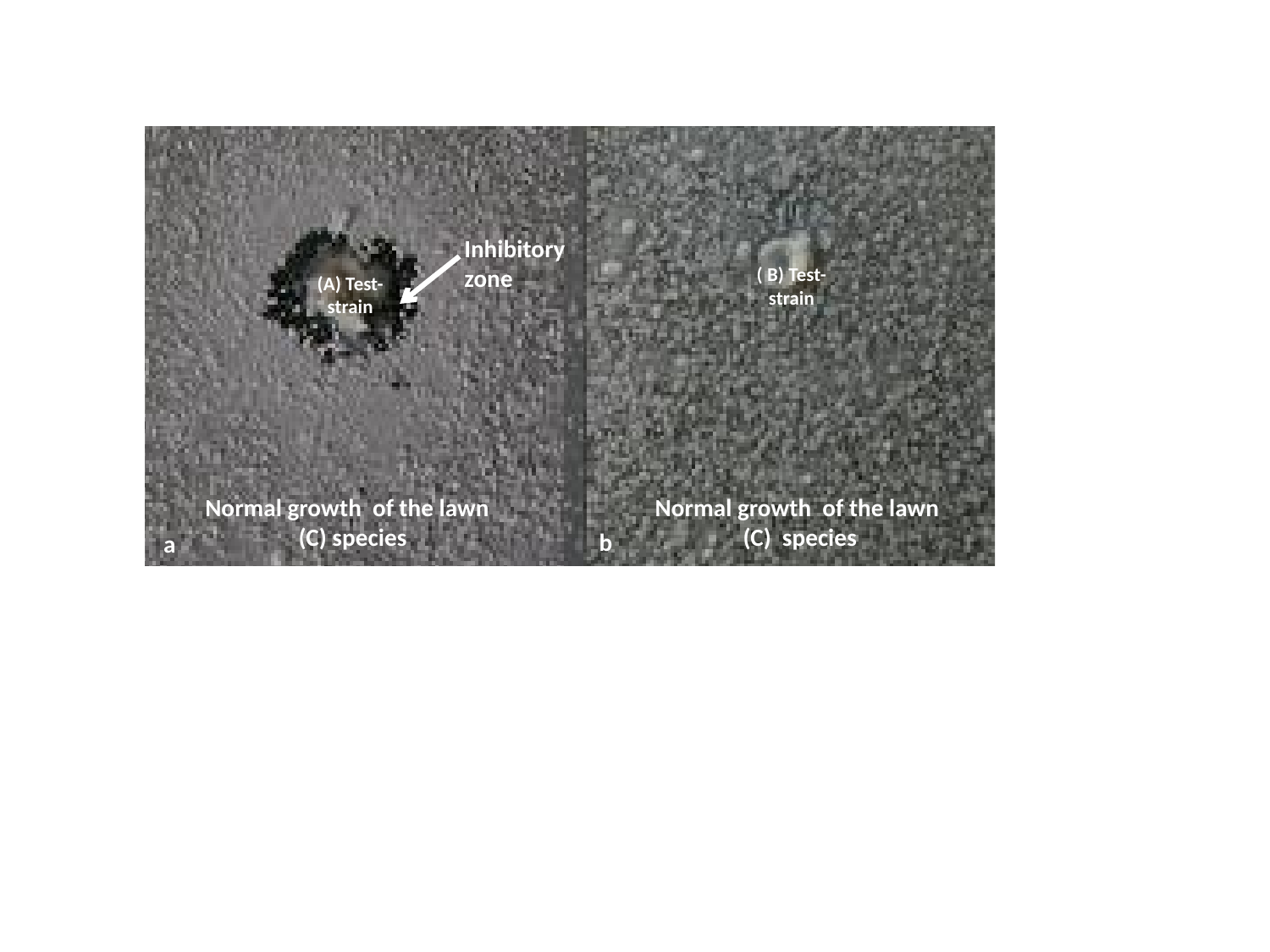

Normal growth of the lawn
 (C) species
(A) Test-strain
a
( B) Test-strain
b
Inhibitory zone
Normal growth of the lawn
(C) species
